# Clinical pathways of breast cancer patients treated in the Federal District, Brazil

**DOI:** 10.11606/S1518-8787.2019053000406

**Published:** 2019-01-18

**Authors:** Ângela Ferreira Barros, Jeniffer Melo de Araújo, Cristiane Murta-Nascimento, Adriano Dias

**Affiliations:** I Escola Superior de Ciências da Saúde. Secretaria de Estado de Saúde do Distrito Federal. Brasília, DF, Brasil; II Universidade Estadual Paulista. Faculdade de Medicina. Programa de Pós-Graduação em Saúde Coletiva. Botucatu, SP, Brasil; III Hospital Alvorada Brasília. Brasília, DF, Brasil; IV Universidade Estadual Paulista. Faculdade de Medicina. Programa de Pós-Graduação em Saúde Coletiva. Departamento de Saúde Pública. Botucatu, SP, Brasil

**Keywords:** Breast Neoplasms, therapy, Time-to-Treatment, Health Care Quality, Access, and Evaluation, Health Status Disparities, Health Services Accessibility, Neoplasias da Mama, terapia, Tempo para o Tratamento, Qualidade, Acesso e Avaliação da Assistência à Saúde, Disparidades nos Níveis de Saúde, Acesso aos Serviços de Saúde

## Abstract

**OBJECTIVE:**

To identify the clinical pathways of women with breast cancer treated in public hospitals, and to analyze the factors that influence the time interval between the first appointment and the start of therapy.

**METHODS:**

A cross-sectional study was conducted with 600 women with breast cancer treated in nine public hospitals in the Brazilian Federal District. Patients were interviewed between September 2012 and September 2014. Simple and multiple logistic regression models were adjusted to evaluate the variables associated with the time interval studied. The most frequent pathway was the one that started in primary care with following care in the therapy service (28.9%). In the multiple adjustment, factors associated to a longer time interval between the first appointment and therapy were: lower family income (OR = 1.89; 95%CI 1.32–2.68), the first appointment in public services (OR = 1.78; 95%CI 1.20–2.64), care in more than two health services in the clinical pathway (OR = 1.71; 95%CI 1.19–2.44); and obtaining the anatomopathological analysis of the biopsy in public services instead of private health services (OR = 1.87; 95%CI 1.29–2.71). Independently, the implementation of specialist appointment scheduling, with care regulation, was associated with a shorter time interval between first appointment and therapy (OR = 0.33; 95%CI 0.16–0.65).

**CONCLUSIONS:**

We observed that multiple pathways were covered by women with breast cancer treated in public services of the Federal District. Socioeconomic iniquities and several aspectos of the pathways covered were associated with a longer time interval between the first appointment and the start of breast cancer therapy.

## RESUMO

**OBJETIVO:**

Conhecer os itinerários terapêuticos de mulheres com câncer de mama tratadas em hospitais públicos, assim como analisar os fatores que influenciam o intervalo de tempo entre a primeira consulta e o início do tratamento.

**MÉTODOS:**

Realizou-se um estudo transversal com 600 mulheres com câncer de mama tratadas em nove hospitais públicos do Distrito Federal, Brasil. As pacientes foram entrevistadas entre setembro de 2012 e setembro de 2014. Foram ajustados modelos de regressão logística simples e múltipla para avaliar as variáveis associadas ao intervalo de tempo estudado. O itinerário mais frequente foi aquele iniciado na atenção primária com atendimento subsequente no serviço de tratamento (28,9%). No ajuste múltiplo, foram associados a um maior tempo entre a primeira consulta e o tratamento: menor renda familiar (OR = 1,89; IC95% 1,32–2,68), realização da primeira consulta em serviços públicos (OR = 1,78; I IC95% 1,20–2,64), atendimento em mais de dois serviços de saúde no itinerário terapêutico (OR = 1,71; IC95% 1,19–2,44); e obtenção da análise anatomopatológica da biópsia em serviços públicos ao invés de serviços de saúde privados (OR = 1,87; IC95% 1,29–2,71). De forma independente, a implementação do agendamento de consulta com especialista, por meio da regulação assistencial, foi associada a um menor intervalo de tempo entre primeira consulta e tratamento (OR = 0,33; IC95% 0,16–0,65).

**CONCLUSÕES:**

Observou-se que múltiplos itinerários foram percorridos pelas mulheres com câncer de mama tratadas em serviços públicos do Distrito Federal. Iniquidades socioeconômicas e diversos aspectos dos itinerários percorridos foram associados a um maior intervalo de tempo entre a primeira consulta e início do tratamento do câncer de mama.

## INTRODUCTION

Breast cancer is the most common neoplasm in women in Brazil and in the Federal District (DF – Distrito Federal), when not considering non-melanoma skin cancer. In 2018, approximately 60 thousand new breast cancer cases were estimated in Brazil, with more than a thousand in DF^1^. Despite the trend towards stabilization in breast cancer mortality rates in Brazil, these rates are growing in states with lower socioeconomic status^2^. This suggests socioeconomic inequities in the access to and use of health services, with delay in diagnosis and/or in the definition and start of the appropriate therapeutic option^2^ ^,^ ^3^.

The existence of properly prepared health services is critical to ensure the access to treatment of confirmed cases, via the early diagnosis and start of therapy in appropriate time^4^. However, the access to tests and to referral services for definitive diagnosis (secondary care) and for therapy (tertiary care) persist as critical parts. This demonstrates the difficulty of coordinating the different levels of care^5^ in a country where mechanisms to organize the flow of care are still lacking^6^ and the access to specialists is unavailable/limited^7^. These frailties may have contributed to most of the delay for diagnosis confirmation^8^ and early treatment^9^ of breast cancer being attributed to the health service, since this delay occurred after the women had their first appointment due to the identification of signs and symptoms suggestive of cancer or changes in tests^8^ ^,^ ^9^.

Studies on the time interval between the first appointment and the start of therapy present different results when conducted in developed or developing countries^10^, considering that each study has its own contexts and peculiarities to analyze the determining factors^11^. Furthermore, it is a consensus that this interval must be as small as possible, since an interval of more than three months between the patient's detection of the symptom and the start of therapy was associated with worse survival rates^12^, and that a late diagnosis leads to more aggressive treatments^10^.

Seeking to expand the access to public services and procedures within the Unified Health System (SUS)^13^ in an orderly, timely, equitable and rational manner, health care networks were organized and resulted in the structuring of regulatory centers; however, their functioning is still incipient^13^ ^,^ ^14^. Moreover, considering that access to health is based on availability, acceptability, capability of payment and information^15^, it is fundamental to consider that several contexts have influence on individuals’ personal behavior when choosing health services^16^. The interaction of different actors, among them patients, health professionals, administrators and health systems, determines the pathway and the wait for therapy^17^.

Thus, given the importance of improving the expedite of health care at different levels, the objective of this study was to identify the clinical pathways of women with breast cancer treated in public hospitals of DF and to investigate its association with the time interval between the first appointment and the start of therapy.

## METHODS

This research is part of a study titled “*Fatores associados ao tempo de acesso para o tratamento do câncer de mama no Distrito Federal, Brasil*”, approved by the Research Ethics Committee of Fundação de Ensino e Pesquisa em Ciências da Saúde da Secretaria de Estado da Saúde do Distrito Federal (SES-DF) (Opinion 99.313).

A cross-sectional study was conducted with women with pathological diagnosis of breast cancer hospitalized for clinical or surgical therapy of this disease, who underwent the first therapy in one of nine public hospitals in the DF and who signed the informed consent form. Prevalent cases of breast cancer and cases that presented metastatic disease prior to the start of therapy were not included in the study.

To calculate the sample size, the number of new cases of breast cancer estimated in the DF for the data collection period (1,800 new cases)^18^, the proportion of new cases treated by private health services (40%)^19^ and the prevalence of delay in starting breast cancer therapy after the first visit, considered as > 3 months time interval (77.6%)^9^, were used as the basis. Thus, it was estimated that 600 cases would be required.

Data collection from consecutive cases was performed between September 2012 and September 2014, using interviews guided by a structured questionnaire and the analysis of the patient's medical record to obtain clinical data.

Women were questioned about the health services in which they were attended to, from the first appointment until the start of breast cancer therapy, in addition to the dates of these visits. Appointment cards, charts and test results were used to help patients remember the dates of their appointments. The place of the first visit was considered the one where the patient was initially examined to evaluate the clinical complaint in her breast, or the unit that requested or performed the tests whose results were considered suspect.

Appointments were considered specialized when the professional was a breast cancer specialist or surgical or clinical oncologist. Non-specialist appointments were those performed by primary care doctors or other specialties found in public outpatient services or in private health services.

Therapy service was the place that performed the surgery or that referred the patient to start chemotherapy. Intermediate services were those where the woman went to an appointment and was then referred to a specialist in the interval between the first non-specialist appointment and the therapy service. The date and place of this appointment were also collected.

Follow-up appointments, appointments with other doctors for preoperative evaluation and appointments that served only for a second opinion were not considered, as well as the dates of the review of the results of anatomopathological examinations.

The types of health services in which the women were attended to were classified as private services or as SUS public services, with the following subclassification: primary health care service (basic health unit or *Estratégia Saúde da Família* [Family Health Strategy]), emergency care service (emergency room), secondary or tertiary care service of other specialties (outpatient clinic without breast cancer specialist, surgical or clinical oncologist) or diagnosis and/or therapy service (outpatient clinic with breast cancer specialist, surgical and/or clinical oncologist).

The date of the surgery or the first chemotherapy session – in the cases that required neoadjuvant therapy – were registered as the date of the start of therapy.

In July 2013, the SES-DF implemented the clinical protocol for appointment scheduling with breast cancer specialists via a care regulation system, which prioritized specialized care for women with imaging tests suspected of malignancy. Previously, women were referred by verbal or written guidance, without the need for diagnostic tests and risk classification, leading to the same type of referral for women with benign or malignant diseases. The effect of this measure on the time interval between the first appointment and the treatment was evaluated by restricting the analysis to women from the DF and whose pathway started in primary care and with subsequent procedures being held in public diagnostic or therapy services. Considering this restriction, the sample for analysis of this independent variable totaled 220 women. Other variables analyzed were the following socioeconomic characteristics: age, self-reported skin color, place of residence (DF or outside the DF), educational level and family income per month expressed in US dollars (1 USD = 2.7 BRL on December 31, 2014).

Data analysis encompassed percentage distribution of categorical variables and measures of central tendency and dispersion of the continuous variables. A simple logistic regression model was used to evaluate the association of predictor variables with a longer time interval between the first appointment and therapy. The response variable was dichotomized according to the median found, due to the low frequency of women with a time interval under three months. Following, a multiple logistic regression model was adjusted by the stepwise forward method. Variables that presented p ≤ 0.25 values in the simple model were tested in the multiple model^20^. Variables were kept in the model when p < 0.05. The software IBM SPSS Statistics v.20.0 was used for statistical analysis.

## RESULTS

Among the 600 participants, the mean age at diagnosis was 53.3 years and most were between 50 and 69 years old (47%), self-reported as being brown (46.4%), lived in the Federal District (65.8%), reported 7.9 (±4.6) years of study on average and had mean monthly family income of USD 770 (±921.93), and USD 502.22 as the median.

We identified a higher prevalence of the pathway with up to two health services, either the location of the first service followed by the first therapy service or the coincidence between the first service and the therapy service (56.2%) (Figure). In 17.1% of the cases, after attending the first health service, there were intermediary appointments in at least one specialized public service for diagnostic investigation before the therapy service.

**Figure:**
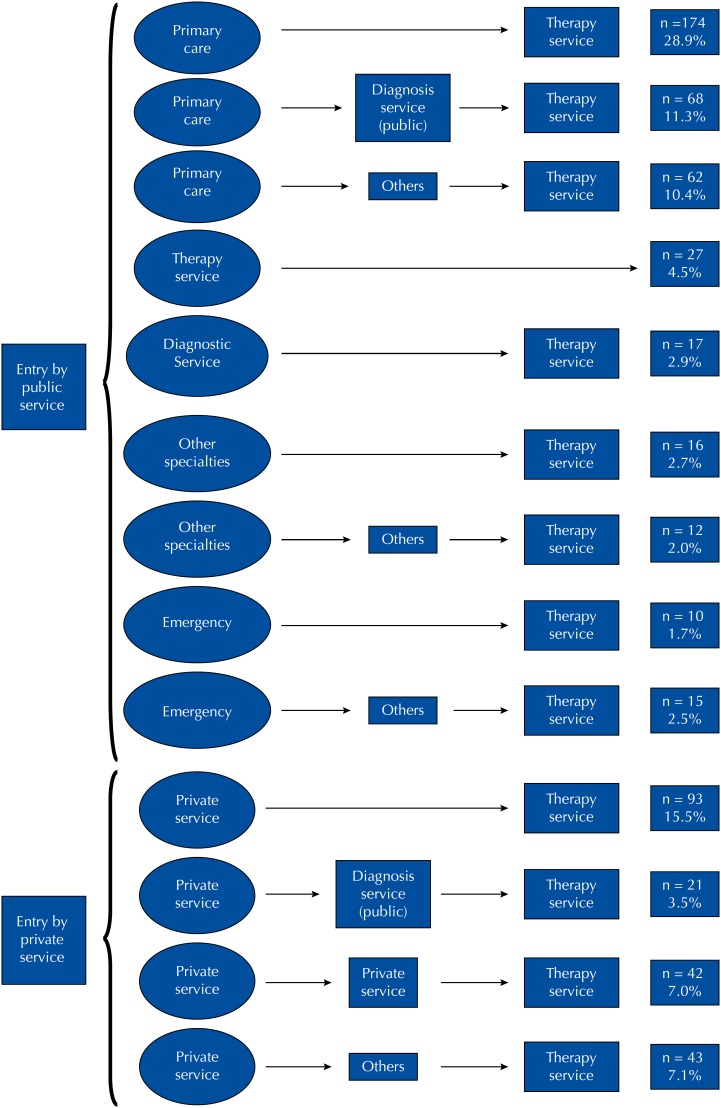
Flowchart of the clinical pathway of 600 women with breast cancer until the start of therapy in nine public hospitals of the Federal District, Brazil, between September 2012 and September 2014.

Primary care was the place where the first appointment was performed more frequently, with private health services in second place (Table 1). Lower percentage of women performed their first appointment directly in specialized secondary or tertiary care service, in secondary or tertiary care service of other specialties or in emergency service (Figure).

**Table 1:** Time interval between the first appointment and the start of breast cancer therapy for 600 women treated in nine public hospitals of the Federal District, Brazil, between September 2012 and September 2014, according to socioeconomic and pathway characteristics.

Variable		n	%	Time interval between the first appointment and therapy	
				Median	Q1–Q3
Place of residence					
	Federal District	395	65.8	153.0	105.0–249.0
	Outside of the Federal District	205	34.2	180.0	102.0–304.0
Self-reported skin color					
	White	226	37.6	150.0	91.5–255.0
	Brown or black	374	62.4	170.0	108.0–284.0
Family income (US$ per month)					
	> 502.20	272	46.7	135.0	91.0–217.0
	≤ 502.20	311	53.3	188.0	120.0–303.5
Educational level (years)					
	> 8	283	52.9	145.0	88.0–246.5
	≤ 8	317	47.1	172.0	117.0–284.0
First appointment location					
	Primary care	304	50.6	191.0	123.0–313.0
	Private health services	199	33.1	133.0	86.0–202.5
	Secondary or tertiary care of another specialty	28	4.7	202.5	107.0–259.0
	Emergency	25	4.2	133.0	101.5–264.5
	Diagnosis and/or therapy services	44	7.4	153.0	86.3–269.8
Number of health services covered					
	≤ 2	337	56.2	149.0	97.0–235.8
	> 2	263	43.8	176.0	118.0–320.0
Use of informal relationships to expedite some service					
	Yes	234	39.0	145.0	90.0–228.0
	No	366	61.0	177.5	117.5–285.5
Cancer staging					
	< IIB	255	42.5	161.0	106.0–280.5
	≥ IIB	345	57.5	158.0	102.0–252.0
Anatomopathological biopsy analysis in private services					
	Yes	260	43.3	135.0	82.0–219.0
	No	340	56.7	187.0	124.8–303.3
Regulation of appointments with specialists^b^					
	Before implantation	176	80.0	196.0	129.0–321.0
	After the implantation	44	20.0	137.0	103.3–191.8

^a^ Differences that correspond to information loss.

b n = 220, referring to women from the Federal District whose pathway began in primary care services with subsequent care in specialized or therapy services.

The first appointment of 13.8% of the women was conducted with specialists. Out of these, 53.7% occurred in public services. Private health services were used to perform at least one test by most patients (97.3%) and were responsible for 43.3% of the anatomopathological biopsy results (Table 1). Considering patients who performed their first appointment in public services, 20.5% obtained the anatomopathological biopsy analysis in private services.

Many participants reported having used informal contacts from their own or from the health professionals who attended to them to achieve a more agile service in the health services (Table 1).

The time interval between the first appointment and the start of the therapy presented 211.8 days mean (175.8 standard deviation) and 160 days median (162.5 interquartile range).

Simple logistic regression is shown in Table 2. Variables that were significantly associated with a longer time interval were brown or black skin color, lower family income, lower educational level, first appointment in public services, more than two health services traversed in the clinical pathway, not using informal relationships to expedite care and having the anatomopathological biopsy analysis done in public services. There was no statistically significant association between the variables residence and cancer staging with the analyzed time interval (Table 2).

**Table 2:** Time interval between the first appointment and the start of breast cancer therapy (≤ or > 160 days) for 600 women treated in nine public hospitals of the Federal District, Brazil, between September 2012 and September 2014, with simple logistic regression using socioeconomic and pathway characteristics.

Variable		Time interval between the first appointment and therapy		OR	95%CI
		≤ 160 days	> 160 days		
Place of residence					
	Federal District	209 (34.8%)	186 (31.0%)	1.00	–
	Outside of the Federal District	94 (15.7%)	111 (18.5%)	1.32	0.94–1.85
Self-reported skin color^b^					
	White	128 (21.3%)	98 (16.3%)	1.00	–
	Brown or black	176 (29.4%)	198 (32.9%)	1.47	1.05–2.05
Family income (US$ per month)^b^					
	> 502.20	164 (28.1%)	108 (18.5%)	1.00	–
	≤ 502.20	127 (21.8%)	184 (31.6%)	2.20	1.58–3.07
Educational level (years)^b^					
	> 8	160 (26.5%)	123 (20.5%)	1.00	–
	≤ 8	143 (23.9%)	174 (29.0%)	1.57	1.14–2.17
First appointment location^b^					
	Private services	127 (21.2%)	72 (11.9%)	1.00	–
	Public services	175 (29.1%)	226 (37.8%)	2.31	1.62–3.28
Number of health services covered^b^					
	≤ 2	181 (29.9%)	156 (25.9%)	1.00	–
	> 2	121 (20.3%)	142 (23.9%)	1.36	0.98–1.88
Use of informal relationships to expedite some service^b^					
	Yes	134 (22.2%)	100 (16.8%)	1.00	–
	No	166 (27.4%)	200 (33.7%)	1.63	1.17–2.28
Cancer staging					
	< IIB	128 (21.3%)	127 (21.2%)		
	≥ IIB	175 (29.2%)	170 (28.3%)	0.98	0.71–1.35
Anatomopathological biopsy analysis in private services^b^					
	Yes	161 (26.8%)	99 (16.5%)	1.00	–
	No	142 (23.7%)	198 (33.0%)	2.27	1.63–3.16
Regulation of appointments with specialists^c^					
	Before the regulation	68 (30.9%)	108 (49.1%)	1.00	–
	After the regulation	29 (13.2%)	15 (6.8%)	0.33	0.16–0.65

a Differences that correspond to information loss.

b Variables that produced p ≤ 0.25 values.

c n = 220, referring to women from the Federal District whose pathway began in primary care services with subsequent care in specialized or therapy services. Due to presenting different amounts when considering other independent variables, this variable was excluded from the adjustment of the multiple model.

Regarding women from the Federal District whose pathway started in primary care, with subsequent treatment in specialized service or therapy service, the time interval was shorter for those referred after the implementation of the clinical protocol in the care regulatory system (Table 2). Due to presenting different amounts of the other independent variables, this variable was not inserted in the multiple model adjustments.

In the adjustment of the multiple logistic regression model, a longer time interval between the first appointment and the start of therapy were found to be associated with women with lower family income, first appointment in public services, treatment in more than two health services in the pathway and the anatomopathological biopsy analysis results made in public services (Table 3).

**Table 3:** Adjustment of the multiple logistic regression model between the response variable time interval between first appointment and therapy (≤ or > 160 days) and associated factors.

Variable	Categories	Adjusted OR	95%CI	p
Family income (US$ per month)	> 502.20	1.00	–	–
	≤ 502.20	1.89	1.32–2.68	< 0.001
First appointment location	Private services	1.00	–	–
	Public services	1.78	1.20–2.64	0.004
Number of health services covered	≤ 2	1.00	–	–
	> 2	1.71	1.19–2.44	0.003
Anatomopathological biopsy analysis in private services	Yes	1.00	–	–
	No	1.87	1.29–2.71	0.001

## DISCUSSION

We observed that women with breast cancer treated in public hospitals in the Federal District followed multiple clinical pathways, the most frequent being the one that started in primary care with subsequent care in the service that performed the therapy. Women with greater social vulnerability took more time to follow these pathways.

Regarding the pathways, our results show the relevance of primary care as a gateway for the population to enter the health system, being the most common means of access to specialized care^21^. Other types of public health services were used less frequently as gateways.

Private health services were the second most frequent place of the first appointment. Other studies that analyzed the pathway until cancer therapy also observed that many patients sought care in private health services^6^ ^,^ ^22^.

These services were also used to perform tests, something that was also reported in other studies with patients treated in the SUS^22^ ^-^ ^24^. In this study, almost all women reported having performed at least one test in private health services, mainly with direct out-of-pocket costs. This may be due to previous experiences of difficulty in accessing the service, from the idea that there is delay in the scheduling of tests^23^ or the insufficient supply of specific procedures^21^ in the SUS. In Brazil, the main activities performed in private health services are appointments with specialists and diagnostic and therapeutic support services. These are bottlenecks of the SUS, in which patients face long waiting lists^25^, with greater difficulties in scheduling tests than scheduling specialized appointments^21^.

Researchers believe that the expansion of SUS primary care coverage lead to an increase in the demand for secondary and tertiary services, making the access to diagnostic services even more difficult^24^. The delay in obtaining the tests affects the access of new patients to specialized services, since it increases the number of return appointments until the case is resolved^26^.

The anatomopathological biopsy analysis was specifically evaluated to exemplify the effect of conducting the tests in private health services on the time to start breast cancer therapy. Approximately half the women performed this test in private services, a factor associated with greater speed for the start of therapy, suggesting that the public health system is still incapable of meeting the demand for specific tests for the early diagnosis of breast cancer. A Brazilian study found a shortage of biopsies when considering the diagnostic need estimated by the number of mammograms suspected of malignancy performed by the SUS^27^.

Given that the access to private services, both for specialized appointments and for tests, is related to higher family income, this variable also remained statistically significant in the multiple adjustment. This shows that higher family income helps patients to cope with critical issues to reach the start of breast cancer therapy in SUS; thus, corroborating the analysis of Santos, according to which the coverage of similar health services, and not only complementary, in the public and private scope, contributes to inequity in the provision, access and use of health services^25^.

Regarding the pathways, there was a greater frequency of patients from primary care services with subsequent treatment in specialized services and/or in the therapy service itself. This pathway is believed to be the most expected and adequate according to the hierarchy of regionalization of health services. Such pathway could be classified as the regime of governmental regulation since it was produced by the legal set that instituted SUS^17^.

Although it presents rational aspects, this regime is crossed by multiple interests and contradictions, being susceptible to arrangements and solutions made by patients, even when referred to predefined pathways^17^. This may have occurred with some women who had appointments in different services between the first appointment and the therapy service. Thus, when considering patients as protagonists facing SUS's limitations, we cannot affirm that the frailties of the services alone have motivated these women to seek different health services. However, as pointed out in other studies^22^ ^,^ ^28^,we cannot rule out that limitations in the effectiveness of the care provided and solution of some services may have favored variations in clinical pathways. Those who included more than two health services were associated with a longer time to start therapy. The referral from primary care to a specialized service capable of performing all diagnostic investigation would decrease the need of going through other services and possibly reduce the time to start therapy, as suggested by a Canadian study^29^.

Strengthening primary care in the ordering of access to other health care levels is critical to optimize the referral, as well as care regulation as an instrument to match supply to demand, prioritizing cases according to the classification of clinical criteria^30^. This study verified that the implementation of care regulation to schedule specialized appointments was associated with shorter time interval to start breast cancer therapy in the DF, a benefit previously pointed by another study^31^. Using this and other strategies that strengthen the interaction between administrators, health workers and patients can also contribute to a more agile access to therapy^31^ and, consequently, to the construction of an equitable health system^30^.

The use of informal relationships with health professionals to facilitate access was common among the participants of this study, which was also reported by other authors^28^ ^,^ ^32^. However, this variable did not remain in the multiple adjustment, indicating that using this mechanism did not interfere in the studied time interval. Nevertheless, we must emphasize that the range of care mediated by a health professional can be interpreted as a privilege, entailing the linkage of access to such professional, weakening the structure and organization of the system itself^32^.

Regarding cancer staging, we found no association with the evaluated time interval, possibly due to the relationship between these variables: clinical signs of breast cancer are more evident for doctors when the disease is at an advanced stage, which can lead to faster a diagnosis and treatment; on the other hand, slow-growing tumors would be more difficult to detect and diagnose^33^ ^,^ ^34^.

As a limitation of the study, we must emphasize that the quantitative approach restricted the possibility of analyzing the women's interpretations on their pathway, as well as the repercussions of other factors – both in the pathway and in the studied time interval. Another possible limitation is the memory bias, for demanding the participants to recall past events. However, we believe that these limitations were minimized by using incident cases with few months elapsed between the experiences and the interview.

In conclusion, in this study, we observed multiple pathways used by women with breast cancer treated in the public services of the DF, with lower socioeconomic level and characteristics of the traversed pathways associated to a longer time interval between the first appointment and the start of breast cancer therapy. The difficulty to perform tests in the health care network for diagnosis and therapy was an important factor for the delay in the start of this therapy. Strengthening strategies such as care regulation was shown to be an effective possibility to improve access to medium and high complexity services and may contribute to reduce iniquities in the health care provided by SUS.

## References

[1] Ministério da Saúde (BR), Instituto Nacional de Câncer José Alencar Gomes da Silva, Coordenação de Prevenção e Vigilância (2017). Estimativa 2018: incidência de câncer no Brasil.

[2] Freitas-Junior R, Gonzaga CMR, Freitas NMA, Martins E, Dardes RCM (2012). Disparities in female breast cancer mortality rates in Brazil between 1980 and 2009. Clinics.

[3] Renck DV, Barros F, Domingues MR, Gonzalez MC, Sclowitz ML, Caputo EL (2014). Equidade no acesso ao rastreamento mamográfico do câncer de mama com intervenção de mamógrafo móvel no sul do Rio Grande do Sul, Brasil. Cad Saude Publica.

[4] Ministério da Saúde (BR) (2015). Instituto Nacional de Câncer José Alencar Gomes da Silva. Diretrizes para a detecção precoce do câncer de mama no Brasil.

[5] Parada R, Assis M, Silva RCF, Abreu MF, Silva MAF, Dias MBK (2008). A política nacional de atenção oncológica e o papel da atenção básica na prevenção e controle do câncer. Rev APS.

[6] Dubow C, Olivo VMF, Ceron MI, Vedootto DO, Dal Moro JS, Oliveira CP (2014). Linha de cuidado como dispositivo para a integralidade da atenção a usuários acometidos por agravos neoplásicos de cabeça e pescoço. Saude Debate.

[7] Yip CH, Cazap E, Anderson BO, Bright KL, Caleffi M, Cardoso F (2011). Breast cancer management in middle-resource countries (MRCs): consensus statement from the Breast Health Global Initiative. Breast.

[8] Rezende MCR, Koch HA, Figueiredo JA, Thuler LCS (2009). Causas do retardo na confirmação diagnóstica de lesões mamárias em mulheres atendidas em um centro de referência do Sistema Único de Saúde no Rio de Janeiro. Rev Bras Ginecol Obstet.

[9] Barros AF, Uemura G, Macedo JLS (2013). Tempo para acesso ao tratamento do câncer de mama no Distrito Federal, Brasil Central. Rev Bras Ginecol Obstet.

[10] Rivera-Franco MM, Leon-Rodriguez E (2018). Delays in breast cancer detection and treatment in developing countries. Breast Cancer (Auckl).

[11] Freitas AGQ, Weller M (2015). Patient delays and system delays in breast cancer treatment in developed and developing countries. Cienc Saude Coletiva.

[12] Richards MA, Westcombe AM, Love SB, Littlejohns P, Ramirez AJ (1999). Influence of delay on survival in patients with breast cancer: a systematic review. Lancet.

[13] Gawryszewski ARB, Oliveira DC, Gomes AMT (2012). Acesso ao SUS: representações e práticas de profissionais desenvolvidas nas Centrais de Regulação. Physis.

[14] Vilarins GCM, Shimizu HE, Gutierrez MMU (2012). A regulação em saúde: aspectos conceituais e operacionais. Saude Debate.

[15] Sanchez RM, Ciconelli RM (2012). Conceitos de acesso à saúde. Rev Panam Salud Publica.

[16] Rabelo MCM, Alves PCB, Souza I (1999). Experiência de doença e narrativa.

[17] Cecílio LCO, Carapinheiro G, Andreazza R, Souza ALM, Andrade MGG, Santiago SM (2014). O agir leigo e o cuidado em saúde: a produção de mapas de cuidado. Cad Saude Publica.

[18] Ministério da Saúde (BR), Instituto Nacional de Câncer José Alencar Gomes da Silva, Coordenação de Prevenção e Vigilância (2011). Estimativa 2012: incidência de câncer no Brasil.

[19] Ministério da Saúde (BR), Agência Nacional de Saúde Suplementar (2014). Informações em Saúde Suplementar TABNET: taxa de cobertura de planos de saúde: assistência médica entre 2012 a 2014, no Distrito Federal, sexo feminino, faixa etária de 30 a 79 anos.

[20] Hosmer DW, Lemeshow S (2005). Applied logistic regression.

[21] Almeida PF, Giovanella L, Mendonça MHM, Escorel S (2010). Desafios à coordenação dos cuidados em saúde: estratégias de integração entre níveis assistenciais em grandes centros urbanos. Cad Saude Publica.

[22] Göttems LBD, Calaça NRS, Souza SFO, Morais TCP, Santana JA, Pires MRGM (2012). Análise da rede de atenção ao câncer de colo uterino a partir da trajetória de usuárias no Distrito Federal-BR. Rev Eletr Gestao Saude.

[23] Soares MC, Mishima SM, Silva RC, Ribeiro CV, Meincke SMK, Corrêa ACL (2011). Câncer de colo uterino: atenção integral à mulher nos serviços de saúde. Rev Gaucha Enferm.

[24] Toledo SRS, Almeida NAM, Souza MR, Minamisava R, Freitas R (2016). Fluxo assistencial de usuárias com câncer de mama na rede pública de atenção à saúde. Rev Eletr Enf.

[25] Santos IS (2011). Evidência sobre o mix público-privado em países com cobertura duplicada: agravamento das iniquidades e da segmentação em sistemas nacionais de saúde. Cienc Saude Coletiva.

[26] Spedo SM, Pinto NRS, Tanaka OY (2010). O difícil acesso a serviços de média complexidade do SUS: o caso da cidade de São Paulo, Brasil. Physis.

[27] Azevedo e Silva G, Bustamante-Teixeira MT, Aquino EML, Tomazelli JG, Santos-Silva I (2014). Acesso à detecção precoce do câncer de mama no Sistema Único de Saúde: uma análise a partir dos dados do Sistema de Informações em Saúde. Cad Saude Publica.

[28] Rangel G, Lima LD, Vargas EP (2015). Condicionantes do diagnóstico tardio do câncer cervical na ótica das mulheres atendidas no Inca. Saude Debate.

[29] Jiang L, Gilbert J, Langley H, Moineddin R, Groome PA (2015). Effect of specialized diagnostic assessment units on the time to diagnosis in screen-detected breast cancer patients. Br J Cancer.

[30] Peiter CC, Lanzoni GMM, Oliveira WF (2016). Regulação em saúde e promoção da equidade: o Sistema Nacional de Regulação e o acesso à assistência em um município de grande porte. Saude Debate.

[31] Baduy RS, Feuerwerker LCM, Zucoli M, Borian JT (2011). A regulação assistencial e a produção do cuidado: um arranjo potente para qualificar a atenção. Cad Saude Publica.

[32] Pontes APM, Cesso RGD, Oliveira DC, Gomes AMT (2009). O princípio de universalidade do acesso aos serviços de saúde: o que pensam os usuários?. Esc Anna Nery Rev Enferm.

[33] Symonds RP (2002). Cancer biology may be more important than diagnostic delay. BMJ.

[34] Crawford SC, Davis JA, Siddiqui NA, Caestecker L, Gillis CR, Hole D (2002). The waiting time paradox: population based retrospective study of treatment delay and survival of women with endometrial cancer in Scotland. BMJ.

